# 1242. Efficacy and Safety of Intravenous Fosfomycin for the Treatment of Multi-resistant Gram Negative Infections

**DOI:** 10.1093/ofid/ofab466.1434

**Published:** 2021-12-04

**Authors:** Tasneem Abdallah, Reem Elajez, Tawheeda Ibrahim, Abeir Alimam, Ali S Omrani

**Affiliations:** 1 Hamad medical corporation, Doha, Ad Dawhah, Qatar; 2 Hamad Medical Corporation, Doha, Ad Dawhah, Qatar

## Abstract

**Background:**

To describe the clinical use, efficacy and safety of intravenous (IV) fosfomycin in the treatment of infections caused by Gram-negative bacteria (GNB).

**Methods:**

Hospitalized patients who received ≥48 hours of IV fosfomycin therapy during September 27, 2017 thru January 31, 2020 were included. The primary outcome was the proportion of subjects with clinical improvement at the end of IV fosfomycin therapy; defined as resolution of baseline signs and symptoms of infection.

**Results:**

Thirty patients were included, of which 19 (63.3%) were males, and the median age was 63.5 years (interquartile range 46─73). Frequent risk factors for GNB infection included hospitalization (23, 76%), receipt of broad-spectrum antibiotics (15, 50%), and surgery (10, 33.3%), all within the preceding 90 days. Urinary tract infection (17, 56.7%) was the most common indication for use of IV fosfomycin, followed by bacteremia (4, 13.3), and skin and soft tissue infections (4, 13.3%). *Kelbsiella pneumoniae* (17, 56.7%), *Escherichia coli* (7, 23.3%) and *Pseudomonas species* (4, 13.3%) were the most common target pathogens. Almost all target pathogens (29, 96.7%) were resistant *in vitro* to ≥1 agent from ≥3 different antimicrobial classes. The primary outcome was achieved in 22 (73.3%) patients. The most frequently observed adverse events were hypokalemia (13, 43.3%) and hypernatremia (7, 23.3%). However, the majority of adverse events were classified as Grade 1 or Grade 2 severity.

Microbiological characteristics

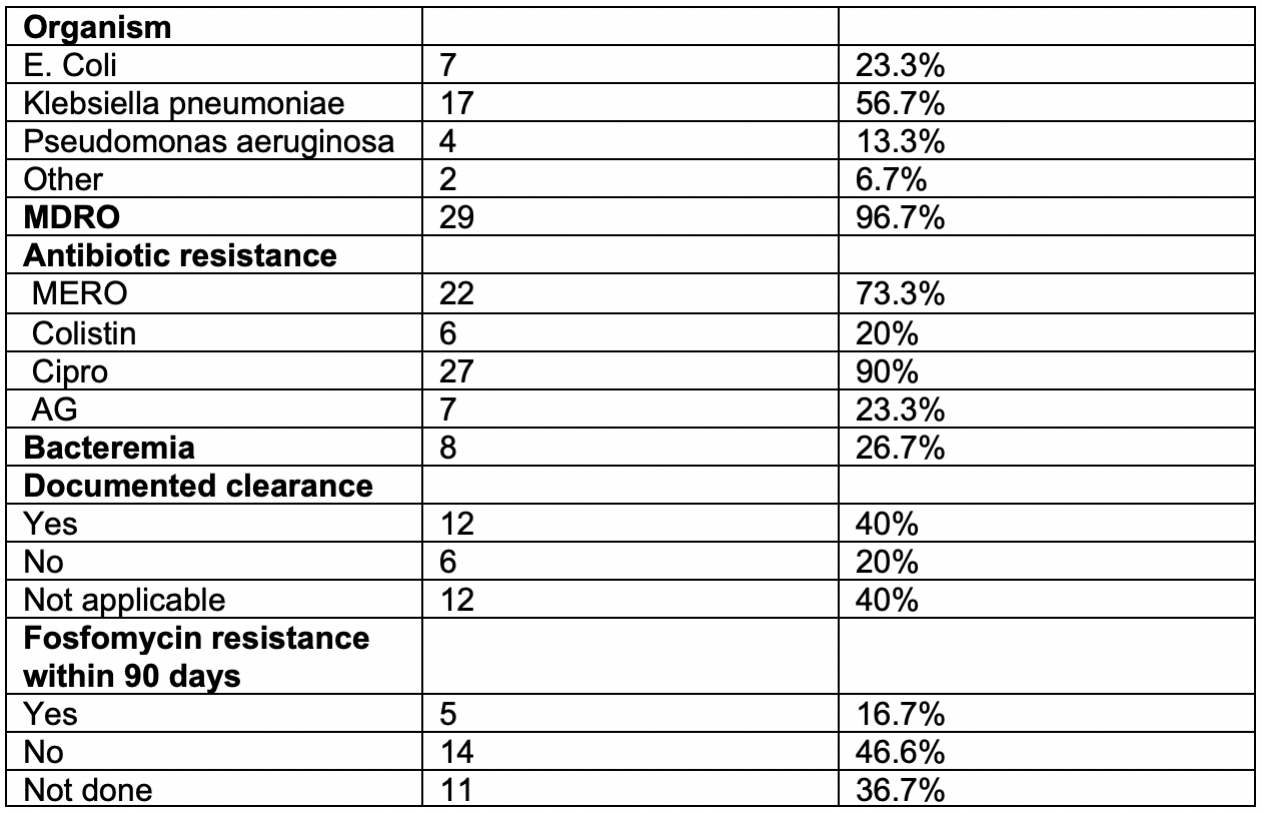

The table describes microbiological characteristics of the isolated organism species, resistance pattern, development of fosfomycin resistance

Management outcomes and safety profile

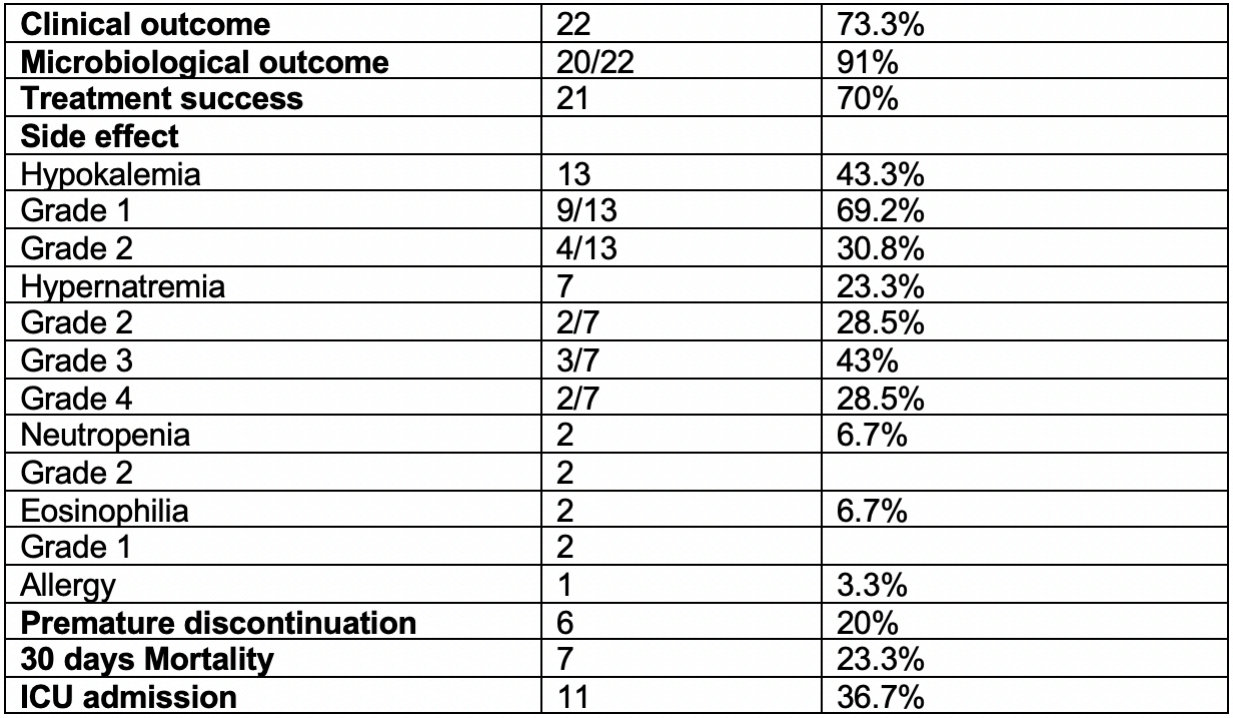

The table describes percentage of primary outcome (clinical success ) along with safety profile and mortality rate

**Conclusion:**

IV fosfomycin is a potentially effective and safe option for the treatment of patient with GNB infections.

**Disclosures:**

**All Authors**: No reported disclosures

